# A Rare Entity Developing After Breast Reconstruction: Pyoderma Gangrenosum

**DOI:** 10.7759/cureus.62361

**Published:** 2024-06-14

**Authors:** Erkan Yanıkoğlu, Can Ekinci, Aydan A Kose

**Affiliations:** 1 Plastic, Reconstructive and Esthetic Surgery, Osmangazi University School of Medicine, Eskişehir, TUR

**Keywords:** wounds, free flaps, mammaplasty, pyoderma gangrenosum, breast

## Abstract

Pyoderma gangrenosum (PG) is a rare and persistent neutrophilic dermatosis with an unknown cause. The condition typically manifests clinically as a pustule or plaque that quickly evolves into a necrotic ulcer with undermined violet-colored margins. A surgical debridement might worsen the disease due to the pathergy phenomenon. This case report presents a 48-year-old woman who underwent a late breast reconstruction with a transverse rectus abdominis myocutaneous flap and was subsequently diagnosed with PG. The report details the delays in the diagnosis and management of the disease, providing a comprehensive account of the course of events.

## Introduction

Breast reconstruction with autologous tissue is the most durable and natural method of breast reconstruction. Furthermore, one of the most popular choices in the field of autologous breast reconstruction is the transverse rectus abdominis myocutaneous (TRAM) flap [1]. Although infection, hematoma, and seroma are the most frequent complications, pyoderma gangrenosum (PG) can be considered rare but possibly one of the worst local complications following breast reconstruction or aesthetic breast surgery [2]. PG is a chronic skin disease with painful, destructive ulcers of unknown cause and is characterized by the pathergy phenomenon, which is the formation or worsening of ulcers even after a minor trauma. The diagnosis is based on clinical features, which include enlarging ulcers with undermined violet-colored margins worsening with trauma. Furthermore, pathological examination showing an acute inflammatory response and neutrophilic infiltration supports the diagnosis [3].

On the other hand, PG, which develops after surgery, is known as postoperative PG (PPG), and it shares the characteristics of neutrophilic dermatoses. Diagnosis may be delayed because PPG mimics necrotizing fasciitis, as both conditions can progress rapidly, causing large wounds. It is a very rare dermatological condition. Unlike common wound infections, the use of debridement in PG or PPG may cause a deterioration of the wound because of pathergy [4]. This report describes a 48-year-old female patient who underwent TRAM flap breast reconstruction of the right mastectomy area and experienced late wound dehiscence in the second postoperative month.

## Case presentation

**Figure 1:**
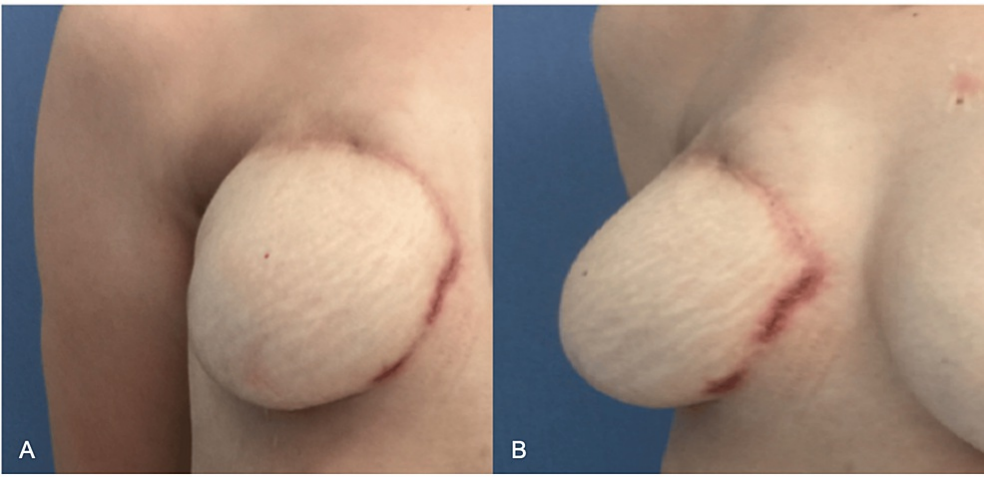
Dehiscence at the suture lines around the second month after TRAM flap surgery, characterized by a necrotic ulcer with undermined violet-colored edges: (A) front view; (B) three-quarter view TRAM, transverse rectus abdominis myocutaneous

After undergoing a right-sided total mastectomy due to invasive ductal carcinoma two years earlier, a 48-year-old patient had breast reconstruction with a TRAM flap. The patient had no known coexisting medical conditions or a family history of autoimmune diseases, which did not raise any concerns during the initial postoperative period. However, complications appeared in the second month after the surgery when the previously closed suture lines dehisced (Figure 1). Since the affected area coincided with Hartrampf’s zone 4 of the TRAM flap, it was initially considered a wound healing problem due to insufficient vascular supply.

**Figure 2:**
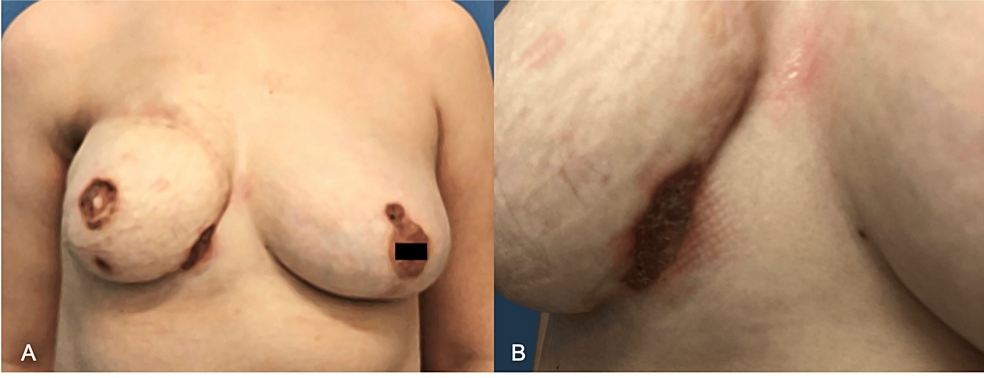
Persistent ulceration around the areola of the left breast 10 months post-surgery, indicative of potential PG: (A) front view; (B) three-quarter view PG, pyoderma gangrenosum

**Figure 3:**
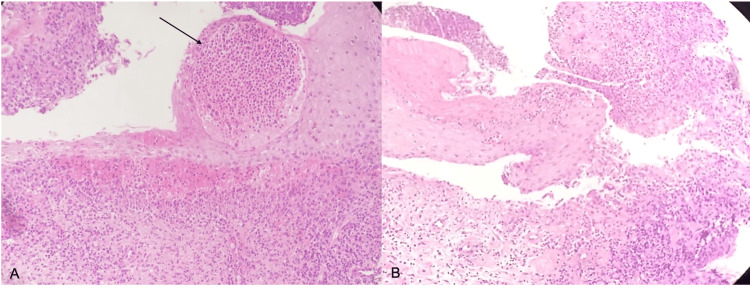
Pathological examination shows abscess-forming neutrophilic dermatosis (A) and an acute inflammatory response with neutrophilic infiltration and loss of epidermis-dermis integrity (B), supporting the diagnosis of PPG PG, postoperative pyoderma gangrenosum

Even after receiving empirical antibiotic therapy and localized wound care, the patient’s symptoms persisted. The wound was then debrided during a scheduled reduction mammoplasty on the left side and concurrent reconstruction of the nipple-areola complex on the right side. Despite meticulous local wound care with various agents such as chlorhexidine gauze dressings, antibiotic ointments, and antiseptic wound cleansers, the wounds did not heal 10 months after the debridement, and ulceration appeared around the areola of the reduced left breast (Figure 2). Consequently, a tissue biopsy for both culture and pathology, as well as blood and urine cultures, was obtained for diagnosis. Pathological examination revealed a nonspecific acute inflammatory response with neutrophilic infiltration and ulceration, indicating a potential diagnosis of PG (Figure 3).

**Figure 4:**
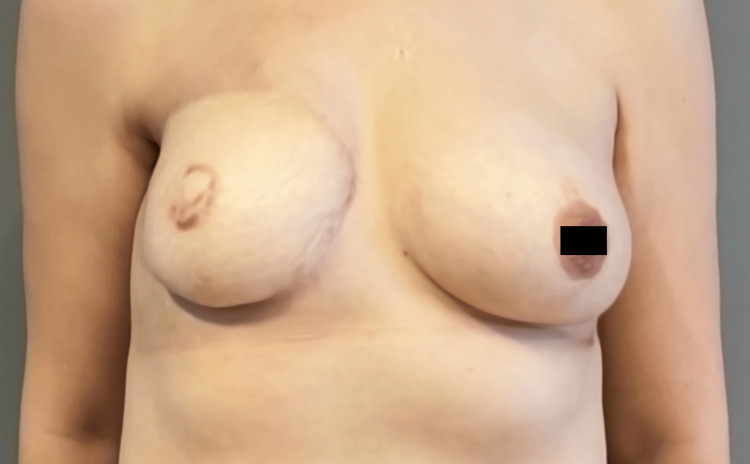
Healed state of the patient showing an acceptable scar four months after the surgery

Following a consultation with dermatology regarding the potential diagnosis of PPG according to the pathology results and clinical findings, the patient began IV methylprednisolone injections at a daily dose of 1 mg/kg. The patient’s lesions stopped progressing just after 48 hours, and re-epithelialization started. Following initial IV methylprednisolone therapy and tapering the dose, IV treatment was eventually stopped after one week, and the treatment continued with oral methylprednisolone for a month. The wound became completely re-epithelialized four months after the treatment, leaving behind an acceptable degree of scarring (Figure 4).

## Discussion

The incidence of PG is incredibly rare, ranging from three to 10 cases per 1,000,000 people [4]. PPG, an even rarer variation of this disease, appears along incision lines after the surgery. In cases where a patient experiences fever, pain, redness, and ulcerations at the site of a surgical incision, the symptoms likely indicate an infection. In such instances, early debridement and the administration of antibiotics are recommended. On the other hand, the incidence of PPG is extremely rare; if present, neither of the treatments would be beneficial, and even the debridement might worsen the situation. However, it has been noted that in only 10% of such cases, debridement was not performed before diagnosis, which could potentially delay the diagnosis of PPG and worsen it. In at least 74% of cases, antibiotics were given, indicating the frequency of misdiagnosis in such situations [5].

PPG is characterized by a rise in neutrophils brought on by surgical trauma as well as pathergy. Since there was no history of any diseases, our case aligns with existing data indicating that PG is associated with systemic underlying diseases in 78% of instances [3], whereas PPG tends to have a lower likelihood of such systemic involvement [4]. Myeloproliferative disorders, rheumatoid arthritis, and inflammatory bowel disease are often associated diseases of PG [6]. The breast, abdomen, and lower extremities are the most frequently affected sites, with women accounting for the majority of cases (72%). Unlike our case, typical PPG onset symptoms show up as local wound dehiscence between five and seven days after surgery [4].

Unlike this case, other similar cases in the literature frequently result in considerations of necrotizing fasciitis or cellulitis due to the rapid progression of symptoms. As such, prompt debridement and IV antibiotic therapy are standard courses of action [7]. In rapidly evolving scenarios, a diagnostic delay of approximately two weeks from symptom onset to confirmation of the diagnosis is common, with the diagnosis relying on negative cultures, specific histopathological and clinical findings, and a positive response to immunosuppressive treatment [8]. On the other hand, because of its relatively slow progression and the location coinciding with Hartrampf’s zone 4 of the TRAM flap, our case was misdiagnosed as a local wound healing problem due to reduced blood flow at first.

Pathergy, a term used to describe the induction or exacerbation of PG at sites of incidental or iatrogenic trauma, is an important phenomenon in the disease course and is frequently mentioned in descriptions of the clinical behavior of PG. Due to pathergy, many researchers continue to argue against the role of surgery in the treatment of PG. Surgery is only considered in select cases, such as necrotic tissue accumulation or exposed vital tissues [9]. It is recommended to consider performing surgical procedures during periods of disease control while providing concomitant systemic immunosuppressive therapy [10]. Therefore, it is advised that perioperative immunosuppressive therapy should be started in patients who have been diagnosed with PPG before considering further debridement or alternative surgical procedures [5].

## Conclusions

PPG is an uncommon dermatosis that mostly affects surgical incisions, is most commonly seen on the breast, and usually advances quickly but can also remain stable, as in this case report. Although PPG commonly develops in the early postoperative period, as in a week in the literature, there can be rare cases in which PPG develops late and progresses slowly, even a couple of months after the surgery.

Due to its particular slow progression and the area of the wound coinciding with the less vascularized region of the flap, we first considered the condition a wound healing problem. Since it did not respond to local wound care and debridement, and pathology showed neutrophilic dermatosis, only after two months was the correct diagnosis of PPG made. Therefore, particularly in patients whose wounds do not regress in spite of appropriate antibiotics and local wound care, with cultures revealing no growth and histopathology revealing neutrophilic inflammation, PPG should be kept in mind as a possible diagnosis, debridement should be avoided, and appropriate immunosuppressive treatment should be considered.

## References

[1] Chang EI (2021). Latest advancements in autologous breast reconstruction. Plast Reconstr Surg.

[2] Gulyas K, Kimble FW (2003). Atypical pyoderma gangrenosum after breast reduction. Aesthetic Plast Surg.

[3] Powell FC, Schroeter AL, Su WP, Perry HO (1985). Pyoderma gangrenosum: a review of 86 patients. Q J Med.

[4] Tolkachjov SN, Fahy AS, Cerci FB, Wetter DA, Cha SS, Camilleri MJ (2016). Postoperative pyoderma gangrenosum: a clinical review of published cases. Mayo Clin Proc.

[5] Dhooghe N, Oieni S, Peeters P, D'Arpa S, Roche N (2017). Post surgical pyoderma gangrenosum in flap surgery: diagnostic clues and treatment recommendations. Acta Chir Belg.

[6] Ashchyan HJ, Butler DC, Nelson CA (2018). The association of age with clinical presentation and comorbidities of pyoderma gangrenosum. JAMA Dermatol.

[7] Kim D, Hur SM, Lee JS, Chin S, Lim CW, Kim Z (2021). Pyoderma gangrenosum mimicking wound infection after breast cancer surgery. J Breast Cancer.

[8] Haag CK, Bacik L, Latour E, Morse DC, Fett NM, Ortega-Loayza AG (2020). Perioperative management of pyoderma gangrenosum. J Am Acad Dermatol.

[9] Reichrath J, Bens G, Bonowitz A, Tilgen W (2005). Treatment recommendations for pyoderma gangrenosum: an evidence-based review of the literature based on more than 350 patients. J Am Acad Dermatol.

[10] Alam M, Grossman ME, Schneiderman PI, Blume RS, Benvenisty AI (2000). Surgical management of pyoderma gangrenosum: case report and review. Dermatol Surg.

